# Hydroxycinnamic acids in cooked potato tubers from *Solanum tuberosum* group Phureja

**DOI:** 10.1002/fsn3.403

**Published:** 2016-07-12

**Authors:** Clara Piñeros‐Niño, Carlos‐Eduardo Narváez‐Cuenca, Ajjamada C. Kushalappa, Teresa Mosquera

**Affiliations:** ^1^ Facultad de Ciencias AgrariasUniversidad Nacional de ColombiaCarrera 30 No.45 ‐ 03, Edificio 500BogotáColombia; ^2^ Departamento de QuímicaUniversidad Nacional de ColombiaCarrera 30 No.45 ‐ 03, Edificio 451BogotáColombia; ^3^ Plant Science DepartmentMcGill UniversitySainte‐Anne‐de‐BellevueQuebecH9X 3V9Canada

**Keywords:** Antioxidants, chlorogenic acid, diploid tuber, isomers, Phenylpropanoid, Potato, *Solanum tuberosum* L. group Phureja

## Abstract

Hydroxycinnamic acids are phenolic compounds and are considered to have health promotion properties due to their antioxidant activity. Potato tubers of 113 genotypes of *Solanum tuberosum* group Phureja belonging to the Colombian Central Collection, landraces of potatoes, and commercial cultivars were evaluated for their hydroxycinnamic acids content. The composition of these compounds was analyzed using cooked tubers in two different agro‐climatic conditions. The genotypes were analyzed for chlorogenic acid, *neo*‐chlorogenic acid, *crypto*‐chlorogenic acid, and caffeic acid by ultrahigh‐performance liquid chromatography (UHPLC). Chlorogenic acid was the major representative and varied between 0.77 to 7.98 g kg^−1^ DW (dry weight) followed by *crypto*‐chlorogenic acid (from 0.09 to 1.50 g kg^−1^ DW). Under moorland agro‐climatic conditions even though the chlorogenic acid levels increased with respect to flatland agro‐climatic conditions, the related isomer *neo*‐chlorogenic acid decreased as compared to flatland conditions. The correlation between chlorogenic acid with the isomers, and with caffeic acid was positive. This study demonstrated that there is a wide variation in hydroxycinnamic acids contents in the germplasm studied, which can be exploited in breeding programs to contribute to human health.

## Introduction

Potato (*Solanum tuberosum* L.) has one of the richest genetic resources of any cultivated plant, (De Haan et al. 2013) and is produced in many environments, under both natural and cultivated conditions (Hawkes 1990). Potato is a staple food in many regions of the world, being the third crop worldwide, and provide important quantities of high‐quality protein, vitamins, minerals, fiber (Clayton and Percival 2000; Andre et al. 2007; Nassar et al. 2012; Peña et al. 2015) phenolic compounds, and carotenoids, with important nutritional and bio‐active value Navarre et al. 2011; Camire et al. 2009.

The nutritional components of potato significantly change among potato genotypes (Andre et al. 2007; Brown 2008). High phenolic contents discovered in domesticated Andean potato landraces have been used in conventional breeding and biotechnological approaches, (Spooner et al. 2005) to develop new cultivars with higher levels of hydroxycinnamic acids (e. g., chlorogenic acid), flavonols (e. g., rutin), and anthocyanins (e. g., petanin) (Shakya and Navarre 2006; Rommens et al. 2008; Navarre et al. 2010; Burgos et al. 2013; Devaux et al. 2014).

The hydroxycinnamic acid concentration is significantly induced following pathogen invasion, and is deposited to enforce the cell walls to arrest pathogen development (Yogendra et al. 2015). In humans, these compounds consumed through the diet are increasingly considered as effective protective agents against the reactive oxygen species (ROS), which are known to be involved in aging and many degenerative diseases (Payyavula et al. 2012; André et al. 2009a). Hydroxycinnamic acids are the main group of phenolic compounds in potato tubers in both diversity and concentration (Shakya and Navarre 2006; Narváez‐Cuenca et al. 2013). Among hydroxycinnamic acids, chlorogenic acid is the most abundant representative. The isomers of chlorogenic acid, *neo*‐chlorogenic acid and *crypto* chlorogenic acid, and caffeic acid are also found in potato tubers (Andre et al. 2007; Malmberg and Theander 1985). Hydroxycinnamic acids content have been found to range widely among taxonomic groups and genotypes of potato. Friedman and Levin (2009) conducted an extensive work in raw tetraploid potato evaluating the presence of phenolic compounds in both skin and flesh by means of HPLC‐DAD‐MS (High‐performance liquid chromatography with diode‐array detection and mass spectrometry detection). The amount of chlorogenic acid ranged in potato skin from 4.4 to 34.0 mg/100 g fresh weight (FW) and in potato flesh from 0.4 to 12.0 mg/100 g FW (Stushnoff et al. 2008). Other authors reported different levels in the chlorogenic acid content comparing different taxonomic groups of potato, Navarre et al. (2011) reported a 20‐times difference in the chlorogenic acid content between the lowest reported (*S. bulbocastanum*, 21.9 mg/100 g dry weight, DW) and highest reported (C097226‐2R/R, 473.0 mg/100 g DW) cultivars.

Narváez‐Cuenca et al. (2012) evaluated phenolic compounds in some Colombian cultivars analyzing raw tubers, belonging to the groups Tuberosum and Phureja, particularly hydroxycinnamic acids and their conjugates. They found a large diversity among cultivars with at least 50 compounds contributing to the hydroxycinnamic profile of potato tubers.

The content of phenolic compounds is highly influenced by environmental conditions in which the plants are grown (André et al. 2009b). Payyavula et al. (2012) showed that the relative proportions of the chlorogenic acid varied among the locations, and that there was up to two‐fold difference in the concentration of chlorogenic acid, while up to 15‐fold difference in the concentration of *neo*‐chlorogenic, and up to 12‐fold difference in the *crypto*‐chlorogenic concentration. Complex traits in plants such as those related with yield, disease resistance, and nutritional contents are highly influenced by environment. Therefore, it is important to know the interaction of genotype by the environmental effect. Some genotypes, however, have genetic stability and under different environments they could show similar responses in the evaluated traits (Flis et al. 2014; Hamouz et al. 2013; Fox et al. 1997).

The Colombian Central Collection (CCC) includes genotypes of *S. tuberosum* group Phureja. This is a cultivated group and it is important because is source of genes for different important agronomical traits and is used for potato breeding programs in Colombia (Mosquera et al., 2013). *S. tuberosum* group Phureja presents a biodiversity center at south of Colombia (Andre et al. 2007; Navarre et al. 2011; Juyó et al. 2015). Group Phureja is mainly conformed by diploid potatoes with a wide phenotypic diversity, for different agronomical and nutritional traits, however, studies about hydroxycinnamic acids are lacking. Phureja has been employed worldwide to understand potato genetics because its ploidy level facilitates its genetic study, also a genotype from the group Phureja was employed for potato genome sequencing (Potato Genome Sequencing Consortium [PGSC], 2011). Some native communities cultivate landraces of potato at south of Colombia in Nariño province. These landraces are preserved by these communities who ascribe different properties from culinary to medicinal uses (Rodríguez and Tinjacá 2015) and some landraces as part of the daily diet (Del Castillo et al. 2014)

The aim of this research was to determine the concentration of the main hydroxycinnamic acids present in cooked tubers of *S. tuberosum* group Phureja belonging to CCC, landraces of potatoes from Nariño province, and commercial cultivars, as well as to evaluate the effect of two different locations on the concentration of the aforementioned compounds in eight selected potato genotypes from group Phureja.

## Materials and Methods

### Plant material

A total of 113 potato genotypes composed of 99 accessions from *S. tuberosum* group Phureja belonging to the CCC and that constitutes the Potato Working Collection for the breeding program at National University of Colombia, nine landraces collected at south of Colombia in Nariño province, and five commercial cultivars were studied in a single agro‐climatic condition. The commercial cultivars analyzed are the current most important cultivars according to the planted area in Colombia.

Potato tubers were sowed in Facatativá locality (2650 m above sea level, masl), a representative potato crop region in Colombia and characterized because it is a flatland. This experiment was conducted during the second semester of 2012 (August to November). Plant material was sowed in sandy loam soil, rich in organic matter, pH 5.02, crop cycle was around 125–130 days. The crop was managed according to the cultural practices used by farmers in Colombia. In field, the 113 genotypes were sowed in alpha design (Patterson et al. 1978) with three repetitions, using three tubers as one experimental unit. Tubers were harvested considering the commercial tuber size (35–60 mm diameter). They were bulked per each cultivar and subsamples of 10 tubers were used in the analysis.

To assess the effect of the agro‐climatic conditions on the hydroxycinnamic acids content, potato tubers were sown in a second locality, with a difference of 800 m of the altitude as compared to the first locality. The second locality was Usme, as moorland agro‐climatic conditions representative (3400 masl), this experiment was conducted in the second semester of 2013 (August to November). Eight genotypes from the CCC were selected because of their yellow or white colored flesh, which is highly appreciated by consumers.

### Chemical and reagents

Methanol, acetonitrile, and acetic acid, all HPLC grade were purchased from J.T. Baker (Phillispsburg, NJ). Chlorogenic acid and caffeic acid standards were purchased from Chromadex (Irvine, CA). *Neo*‐chlorogenic and *crypto*‐chlorogenic acids were purchased from PhytoLab (Vestenbergsgreuth, Germany). All standards were prepared as stock solutions at 1.00 mg/mL in methanol and stored at −20°C until use. HPLC‐grade water was obtained using a Millipore system (Billerica, MA).

### Conditioning of samples

Potato tubers were washed with tap water to remove the soil and rinsed with distilled water. Whole tubers with skin were cooked in water, (Im et al. 2008) using a standardized time determined experimentally to obtain a palatable product according to the tuber size (Peña et al. 2015). Cooked potato tubers were cut into slices of 0.8 cm, frozen at −20°C, freeze‐dried, ground to a particle size lower than 0.2 mm, and finally stored in hermetically sealed bags under dark conditions in a desiccator.

### Hydroxycinnamic acids analysis

#### Extraction

Potato powder (50 mg) was homogenized with acidified aqueous methanol (acetic acid:methanol:water, 0.1:50:50, *v/v/v*) in an orbital shaker (112 g/10 min) at 4°C and centrifuged (22,000*g*/5 min/4°C). Extraction was performed five times. The five supernatants were mixed together, filtered using disposable nylon syringe filter units (0.2 *μ*m, Thermo Scientific, Waltham MA), flushed with nitrogen gas, and stored at −20°C until further analyses.

### Identification and quantification of major hydroxycinnamic acids by UHPLC coupled to a DAD

Identification and quantification of the main hydroxycinnamic acids was done in a Dionex Ultimate 3000 ultrahigh‐performance liquid chromatography (UHPLC) system (Thermo Scientific) coupled to a diode array detector (DAD). Potato extracts (5 *μ*L) were analyzed using a Hypersil gold RP‐C18 column (150 mm × 2.1 mm, 1.9 *μ*m particle size, Thermo Scientific) coupled to a precolumn (10 mm × 2 mm, 3 *μ*m, Thermo Scientific). Temperature of the precolumn and column was set to 30°C. The mobile phases were water/acetonitrile/acetic acid (99:1:0.1, *v/v/v*) (Eluent A) and acetonitrile/acetic acid (100:0.1, *v/v*) (Eluent B). The program used was 0–5 min, isocratic at 0% B; 5–23 min, linear gradient from 0 to 60% B; 23 to 24 min, linear gradient from 60 to 100% B; 24 to 27 min, isocratic at 100% B; 27 to 28 min, linear gradient from 100 to 0% B; and 28 to 35 min, isocratic at 0% B. The solvent flow rate was constant at 400 *μ*L/min. Chromatograms were recorded at 320 nm.

The standards employed were chlorogenic acid (5‐*O*‐caffeoylquinic acid, ChA), its isomers *neo*‐chlorogenic acid (3‐*O*‐caffeoylquinic acid, *neo*‐ChA) and *crypto*‐chlorogenic acid (4‐*O*‐caffeoylquinic acid, *crypto*‐ChA), and caffeic acid (3,4‐dihydroxycinnamic acid, CaA). According to the high number of samples that were analyzed, every 10 samples a control sample (CCC019) was injected to check the stability of the chromatographic system, both retention time (Relative standard variation lower than 0.01 %) and concentration (Relative standard variation lower than 2.0%) of chlorogenic acid in CCC019 were evaluated. The identification was done by comparison of retention times, UV spectrum, and behavior after spiking of an extract from the genotype CCC019 with the standards. The quantification was performed based on the external standard method with calibration curves of standard solutions ranging from 0.02 mg/L to 20 mg/L (*n* = 5, in all cases *r* ^2 ^≥ 0.999). ChA, *neo*‐ChA, and *crypto*‐ChA were quantified by means of authentic ChA calibration curves. CaA was quantified based on calibration curves made with authentic caffeic acid. Results were expressed as grams per kilograms of boiled potato dry weight (g kg^−1^ DW).

### Statistical analysis

To evaluate the concentration of the main hydroxycinnamic acid compounds, the data were obtained using three biological repetitions. Summary statistics are reported as mean and standard deviation. Principal component analysis (PCA) and hierarchical clustering was done using the packages Ade4, (Dray and Dufour 2007) FactoMineR, (Lê et al. 2008) and Performance Analytics,(Peterson et al. 2015) statistical packages for use with R^®^ version 3.1.1 (R Development Core Team 2013) for Windows.

## Results and Discussion

**Figure 1:**
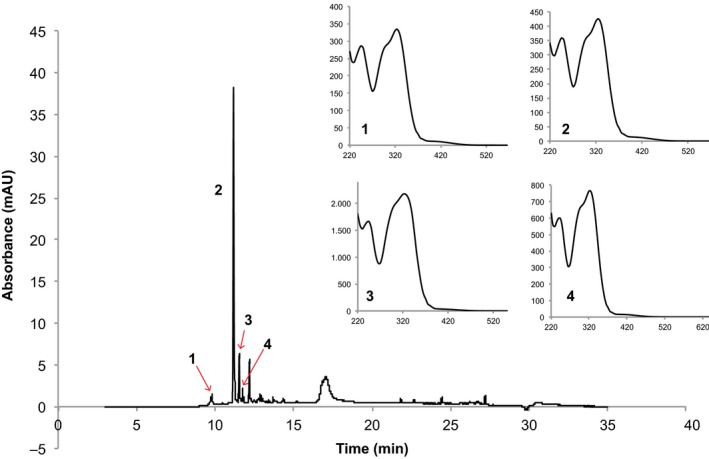
Chromatographic profile of hydroxycinnamic acids in the genotype CCC102 recorded at 320 nm. Retention times were: 1: *neo*‐ChA (9.75 min); 2: ChA (11.14 min); 3: *crypto*‐ChA (11.57 min); 4: CaA (11.78 min). Inserts correspond to the UV spectrum of chromatographic peaks 1–4 from CCC102 [*x* axis: wavelength (nm), *y* axis milliunits of absorbance (mAU)] ChA, chlorogenic acid; CaA, caffeic acid.

A large variation on hydroxycinnamic acid compounds was observed among the 113 studied potato genotypes. The retention time for *neo*‐ChA was 9.75 min, for ChA was 11.14 min, for *crypto*‐ChA was 11.57 min, and for CaA was 11.78 min. These peaks are presented in the chromatograms of the genotype CCC102 (Fig. 1). The mentioned compounds had maximum absorption at 325 nm, with a shoulder at 300–315 nm for ChA and its isomers and 322 nm maxima absorption with a shoulder at 241 mm for CaA (Fig. 1). UV spectrum of the annotated compounds was coincident with those of authentic standards.

### Variation in the concentration of hydroxycinnamic acids among genotypes

The hydroxycinnamic acid contents are shown in Table 1. Both type and concentration of hydroxycinnamic acids varied among genotypes. The concentration of ChA, *neo*‐ChA, *crypto*‐ChA, and CaA showed high variation among genotypes, mainly in *crypto*‐ChA and *neo*‐ChA (ranging from 0.02 to 1.50 g kg^‐1^ DW, 75.0‐fold change and from 0.02 to 1.12, 56.0‐fold change, respectively), whereas CaA and ChA presented lower variation (ranging from and 0.01 to 0.13 g kg^−1^ DW, 13.0‐fold change, and 0.77 to 7.98 g kg^−1^ DW, 10.4‐fold change, respectively). ChA was the hydroxycinnamic acid found in higher concentration. ChA presented variations between 0.77 to 5.87 g kg^−1^ DW in the CCC, between 0.96 to 7.98 g kg^−1^ DW in the Nariño landraces, and between 1.02 to 1.90 g kg^−1^ DW in the commercial cultivars (Table 1). The differences observed in tubers of the three Phureja groups analyzed (CCC, Nariño landraces, and commercial cultivars), suggest the action of different control elements regulating the isomers concentration. According to Friedman, nongenetic sources of variation, and environmental action, can contribute to the wide range of variation observed for hydroxycinnamic acids content in potato tubers (Friedman 1997). In this sense, Payyavula et al. (2012) found that hydroxycinnamic acids response to form ChA and their isomers are dependent of environment effects (such as light and temperature) and suggested an effect toward *hydroxycinnamoyl‐CoA quinate transferases gene* (*HQT*), this gene controls mainly the synthesis and it is influenced by light, temperature, and biotic stress.

**Table 1:** Concentration of hydroxycinnamic compounds in cooked tubers from 113 potato genotypes (*Solanum tuberosum* group Phureja), Facatativá locality

Genotype	Chlorogenic acid (ChA)	*neo‐*ChA	*crypto‐*ChA g kg^−1 ^DW	Caffeic acid
CCC002 *	2.88 ± 0.77	0.47 ± 0.16	0.71 ± 0.34	0.06 ± 0.03
CCC003*	1.41 ± 0.32	0.10 ± 0.07	0.59 ± 0.28	0.01 ± 0.01
CCC004 *	4.53 ± 1.05	0.31 ± 0.20	0.96 ± 0.03	0.05 ± 0.01
CCC005*	2.16 ± 0.60	0.23 ± 0.06	0.60 ± 0.15	0.05 ± 0.02
CCC006*	2.62 ± 1.05	0.14 ± 0.12	0.66 ± 0.40	0.06 ± 0.03
CCC007*	2.51 ± 1.05	0.11 ± 0.05	0.44 ± 0.03	0.02 ± 0.01
CCC008*	0.88 ± 0.28	0.11 ± 0.06	0.21 ± 0.19	0.06 ± 0.03
CCC009 *	2.48 ± 0.67	0.14 ± 0.04	0.77 ± 0.08	0.06 ± 0.01
CCC011 *	2.95 ± 0.21	0.22 ± 0.07	0.80 ± 0.66	0.05 ± 0.04
CCC013*	2.00 ± 1.57	0.08 ± 0.03	0.30 ± 0.12	0.04 ± 0.02
CCC014*	2.76 ± 0.56	0.10 ± 0.03	0.75 ± 0.19	0.06 ± 0.02
CCC015*	1.33 ± 0.51	0.09 ± 0.04	0.34 ± 0.12	0.03 ± 0.01
CCC016*	1.16 ± 0.21	0.05 ± 0.03	0.12 ± 0.10	0.03 ± 0.01
CCC017*	1.49 ± 0.50	0.17 ± 0.08	0.53 ± 0.19	0.02 ± 0.02
CCC019*	1.69 ± 0.22	0.25 ± 0.11	0.77 ± 0.07	0.06 ± 0.58
CCC020*	1.11 ± 0.15	0.03 ± 0.01	0.17 ± 0.06	0.03 ± 0.02
CCC021*	3.11 ± 0.48	0.22 ± 0.01	0.72 ± 0.05	0.12 ± 0.02
CCC023 *	2.58 ± 0.13	0.18 ± 0.02	1.01 ± 0.09	0.05 ± 0.01
CCC024*	2.81 ± 0.49	0.16 ± 0.12	0.60 ± 0.09	0.06 ± 0.04
CCC027*	1.33 ± 0.54	0.14 ± 0.10	0.43 ± 0.17	0.02 ± 0.01
CCC031*	2.18 ± 0.07	0.18 ± 0.06	0.46 ± 0.12	0.04 ± 0.02
CCC032*	2.44 ± 0.20	0.10 ± 0.02	0.45 ± 0.09	0.03 ± 0.01
CCC033*	0.93 ± 0.41	0.03 ± 0.03	0.14 ± 0.15	0.05 ± 0.04
CCC034*	2.36 ± 0.42	0.04 ± 0.02	0.16 ± 0.04	0.05 ± 0.02
CCC035*	1.02 ± 0.28	0.16 ± 0.01	0.24 ± 0.19	0.03 ± 0.01
CCC036*	1.05 ± 0.15	0.18 ± 0.06	0.26 ± 0.22	0.03 ± 0.01
CCC037*	2.42 ± 0.37	0.23 ± 0.06	0.75 ± 0.16	0.06 ± 0.03
CCC038*	2.68 ± 1.92	0.10 ± 0.13	0.43 ± 0.26	0.03 ± 0.03
CCC040*	2.10 ± 0.51	0.09 ± 0.04	0.39 ± 0.06	0.04 ± 0.01
CCC041*	2.27 ± 0.50	0.08 ± 0.05	0.35 ± 0.08	0.07 ± 0.02
CCC042*	1.69 ± 0.51	0.10 ± 0.07	0.22 ± 0.18	0.03 ± 0.02
CCC043*	3.36 ± 0.96	0.10 ± 0.02	0.61 ± 0.18	0.08 ± 0.04
CCC044*	2.23 ± 0.71	0.28 ± 0.13	0.80 ± 0.36	0.01 ± 0.01
CCC045*	1.21 ± 0.15	0.10 ± 0.25	0.35 ± 0.06	0.03 ± 0.01
CCC047*	2.25 ± 0.51	0.25 ± 0.07	0.71 ± 0.05	0.05 ± 0.01
CCC051*	2.11 ± 1.14	0.24 ± 0.19	0.58 ± 0.53	0.05 ± 0.05
CCC053*	2.27 ± 0.14	0.13 ± 0.14	0.60 ± 0.08	0.09 ± 0.03
CCC056*	2.63 ± 0.92	0.28 ± 0.24	0.66 ± 0.02	0.06 ± 0.01
CCC057*	1.59 ± 0.43	0.12 ± 0.09	0.42 ± 0.19	0.03 ± 0.01
CCC059*	2.22 ± 0.45	0.33 ± 0.16	1.03 ± 0.16	0.09 ± 0.02
CCC061*	2.71 ± 0.78	0.09 ± 0.05	0.40 ± 0.16	0.09 ± 0.03
CCC062*	0.97 ± 0.31	0.07 ± 0.06	0.09 ± 0.05	0.02 ± 0.02
CCC063*	1.45 ± 0.55	0.24 ± 0.10	0.63 ± 0.13	0.06 ± 0.02
CCC065*	2.51 ± 1.36	0.18 ± 0.07	0.73 ± 0.24	0.06 ± 0.03
CCC066*	4.96 ± 0.32	0.27 ± 0.09	0.91 ± 0.11	0.060.01
CCC067*	1.61 ± 0.18	0.08 ± 0.05	0.40 ± 0.06	0.03 ± 0.02
CCC069*	4.25 ± 0.97	0.12 ± 0.07	0.59 ± 0.24	0.07 ± 0.02
CCC070*	1.91 ± 0.16	0.06 ± 0.02	0.28 ± 0.08	0.02 ± 0.02
CCC071*	1.33 ± 0.41	0.07 ± 0.01	0.37 ± 0.09	0.03 ± 0.02
CCC072*	2.89 ± 1.02	0.07 ± 0.05	0.60 ± 0.14	0.07 ± 0.02
CCC073*	2.26 ± 0.92	0.09 ± 0.05	0.16 ± 0.13	0.03 ± 0.00
CCC074*	1.94 ± 0.80	0.08 ± 0.03	0.29 ± 0.06	0.03 ± .01
CCC076*	2.82 ± 0.19	0.18 ± 0.09	0.53 ± 0.11	0.02 ± 0.02
CCC079 *	3.23 ± 1.19	0.14 ± 0.11	0.75 ± 0.43	0.07 ± 0.07
CCC080*	1.65 ± 0.16	0.08 ± 0.05	0.27 ± 0.12	0.04 ± 0.02
CCC081*	5.81 ± 2.66	0.20 ± 0.13	0.95 ± 0.46	0.02 ± 0.02
CCC083 *	3.16 ± 1.19	0.18 ± 0.09	0.76 ± 0.14	0.00 ± 0.01
CCC086 *	2.48 ± 1.51	0.18 ± 0.13	0.81 ± 0.14	0.06 ± 0.03
CCC087*	1.00 ± 0.19	0.05 ± 0.04	0.29 ± 0.11	0.04 ± 0.01
CCC088*	2.01 ± 1.94	0.09 ± 0.05	0.29 ± 0.08	0.01 ± 0.01
CCC089 *	2.54 ± 0.99	0.11 ± 0.08	0.69 ± 0.47	0.07 ± 0.04
CCC091*	3.14 ± 2.26	0.08 ± 0.05	0.44 ± 0.41	0.03 ± 0.02
CCC093*	0.93 ± 0.58	0.06 ± 0.02	0.30 ± 0.15	0.03 ± 0.03
CCC094*	2.23±1.23	0.12 ± 0.04	0.47 ± 0.10	0.02 ± 0.02
CCC096*	1.53 ± 0.42	0.14 ± 0.05	0.34 ± 0.12	0.05 ± 0.02
CCC098*	1.61 ± 0.78	0.06 ± 0.01	0.26 ± 0.03	0.03 ± 0.01
CCC099*	2.99 ± 0.34	0.15 ± 0.13	0.62 ± 0.10	0.05 ± 0.01
CCC100 *	4.97 ± 1.87	0.24 ± 0.18	0.61 ± 0.54	0.02 ± 0.01
CCC101*	1.11 ± 0.45	0.04 ± 0.03	0.17 ± 0.04	0.02 ± 0.00
CCC102*	2.20 ± 0.33	0.26 ± 0.16	0.99 ± 0.26	0.05 ± 0.02
CCC103*	1.69 ± 0.68	0.05 ± 0.03	0.26 ± 0.08	0.02 ± 0.01
CCC104*	1.73 ± 1.39	0.17 ± 0.24	0.59 ± 0.49	0.01 ± 0.01
CCC106*	3.63 ± 0.65	0.19 ± 0.10	0.82 ± 0.04	0.06 ± 0.01
CCC108*	1.55 ± 0.94	0.05 ± 0.01	0.35 ± 0.07	0.05 ± 0.05
CCC109*	2.72 ± 0.65	0.12 ± 0.06	0.84 ± 0.10	0.04 ± 0.03
CCC110*	2.68 ± 1.66	0.12 ± 0.11	0.44 ± 0.16	0.08 ± 0.09
CCC112*	0.77 ± 0.09	0.03 ± 0.01	0.15 ± 0.06	0.02 ±0.02
CCC113*	1.05 ± 0.47	0.06 ± 0.03	0.21 ± 0.08	0.03 ± 0.02
CCC115 *	5.87 ± 2.40	0.43 ± 0.14	1.50 ± 0.10	0.06 ± 0.00
CCC116*	1.18 ± 0.47	0.04 ± 0.02	0.26 ± 0.07	0.01 ± 0.01
CCC117*	4.53 ± 1.77	0.17 ± 0.07	0.95 ± 0.18	0.10 ± 0.04
CCC120*	1.20 ± 0.23	0.07 ± 0.03	0.30 ± 0.05	0.08 ± 0.02
CCC121*	2.45 ± 0.50	0.13 ± 0.08	0.73 ± 0.16	0.05 ± 0.01
CCC122*	2.83 ± 1.31	0.11 ± 0.08	0.65 ± 0.03	0.05 ± 0.01
CCC123*	1.47 ± 0.11	0.05 ± 0.04	0.29 ± 0.04	0.04 ± 0.02
CCC124*	2.73 ± 0.17	0.25 ± 0.05	0.81 ± 0.05	0.04 ± 0.01
CCC125*	3.79 ± 0.70	0.19 ± 0.21	0.87 ± 0.18	0.05 ± 0.01
CCC126*	1.61 ± 0.51	0.09 ± 0.02	0.02 ± 0.00	0.03 ± 0.01
CCC127*	2.58 ± 0.72	0.09 ± 0.06	0.14 ± 0.22	0.01 ± 0.01
CCC128*	2.94 ± 0.44	0.17 ± 0.09	0.68 ± 0.08	0.04 ± 0.01
CCC129*	1.84 ± 0.62	0.05 ± 0.01	0.38 ± 0.09	0.09 ± 0.06
CCC133*	1.44 ± 0.73	0.05 ± 0.05	0.34 ± 0.30	0.03 ± 0.03
CCC136*	1.98 ± 0.59	0.10 ± 0.04	0.40 ± 0.17	0.05 ± 0.01
CCC137*	1.45 ± 0.42	0.07 ± 0.03	0.15 ± 0.04	0.04 ± 0.01
CCC140*	3.83 ± 1.08	0.17 ± 0.15	0.55 ± 0.32	0.04 ± 0.03
CCC141*	1.36 ± 0.43	0.05 ± 0.03	0.67 ± 0.22	0.04 ± 0.01
CCC142*	3.83 ± 0.68	0.22 ± 0.15	1.04 ± 0.20	0.13 ± 0.02
CCC144*	1.44 ± 1.05	0.06 ± 0.03	0.22 ± 0.14	0.03 ± 0.02
CCC145*	2.07 ± 0.45	0.04 ± 0.00	0.17 ± 0.07	0.03 ± 0.02
CrGN*	1.02 ± 0.01	0.04 ± 0.00	0.09 ± 0.00	0.02 ± 0.00
CrCol*	1.89 ± 0.10	0.03 ± 0.01	0.12 ± 0.01	0.03 ± 0.01
CrLat*	1.36 ± 0.11	0.02 ± 0.00	0.08 ± 0.01	0.01 ± 0.01
CrGal*	1.17 ± 0.10	0.03 ± 0.01	0.08 ± 0.03	0.02 ± 0.00
CrPa*	1.90 ± 0.05	0.05 ± 0.00	0.14 ± 0.01	0.07 ± 0.00
Cn005*	1.71 ± 0.15	0.38 ± 0.13	0.24 ± 0.03	0.02 ± 0.00
Cn009*	3.61 ± 0.12	0.55 ± 0.01	0.66 ± 0.02	0.02 ± 0.00
Cn008*	4.48 ± 0.20	0.90 ± 0.10	0.83 ± 0.05	0.03 ± 0.00
Cn010*	3.71 ± 0.75	1.12 ± 0.46	1.08 ± 0.43	0.03 ± 0.01
Cn011*	7.98 ± 0.41	0.84 ± 0.02	1.20 ± 0.11	0.04 ± 0.01
Cn029*	6.69 ± 0.22	0.45 ± 0.03	0.72 ± 0.04	0.02 ± 0.00
Cn042*	0.96 ± 0.05	0.07 ± 0.00	0.08 ± 0.00	0.01 ± 0.00
Cn124*	2.73 ± 0.08	0.65 ± 0.12	0.57 ± 0.03	0.02 ± .00
Cn148*	3.13 ± 0.03	0.38 ± 0.03	0.28 ± 0.00	0.04 ± 0.00
Mean	2.30	0.14	0.51	0.05
SD	1.26	0.19	0.30	0.02

Asterisk color indicates the cluster to which each genotype belongs in the hierarchical clustering analysis (Fig. 3). Black is Cluster I, red is Cluster II, green is Cluster III, blue is cluster IV. Underlined genotypes have red or purple flesh. Genotypes from the Colombian Central Collection are coded as CCC; landraces from Nariño province are coded as Cn; commercial cultivars are coded as Cr (CrGn, Criolla Guaneña; CrGal, Criolla Galeras; CrLat, Criolla Latina; CrCol, Criolla Colombia; CrPa, Criolla Paisa). DW, dry weight. Data are presented as mean (*n* = 3) ± SD.

### Correlations among hydroxycinnamic acids contents

**Figure 2:**
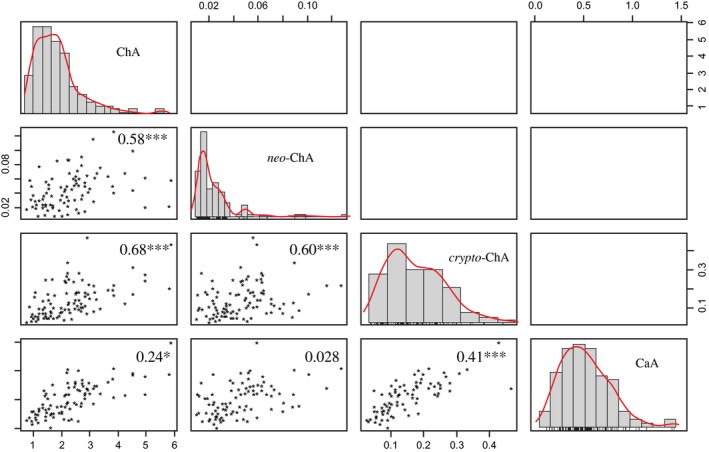
Correlation and distribution of concentrations (Histograms with kernel density overlay) of hydroxycinnamic acids among cooked tubers of *Solanum tuberosum* group Phureja genotypes, sowed in Facatativá locality (2650 masl). Two‐dimensional scatter plots indicate hydroxycinnamic compounds correlation performance. Numbers indicate the absolute correlation values. Axis scales are in g kg^−1^ dry weight. *P < 0.05, ***P < 0.001. ChA, chlorogenic acid; CaA, caffeic acid.

The correlation among hydroxycinnamic acids contents is showed in scatter plots between each pair of variables (Fig. 2). All correlation coefficients between hydroxycinnamic acids were positive. Strong correlations between ChA and its isomers *neo*‐ChA (*r* = 0.58) or *crypto*‐ChA (*r* = 0.68) were found, as well as between *neo*‐ChA and *crypto*‐ChA (*r* = 0.60) and between *crypto*‐ChA and CaA (*r* = 0.41). Furthermore, while a weak correlation between ChA and CaA was found (*r* = 0.24), no correlation between *neo*‐ChA and CaA (*r* = 0.028) was observed. Our results agree with report from Payyavula et al. (2015). According to these authors ChA was the major phenolic compound in potato tubers and ChA had a positive strong correlation with both *neo*‐ChA and *crypto*‐ChA. In Solanaceous species the correlation among different hydroxycinnamic acids might reflect regulatory mechanisms balancing the branches of the phenylpropanoid pathways (Plazas et al. 2013; Clé et al. 2008) as ChA can be mobilized to form downstream phenylpropanoid products (André et al. 2009b).

### Principal components analysis and hierarchical cluster analysis

**Figure 3:**
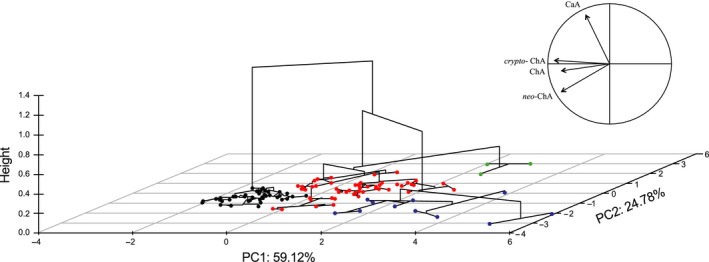
Hierarchical clustering analysis, over two principal components, of 113 genotypes of *Solanum tuberosum* group Phureja cooked tubers. Plot shows four groups. Cluster I (black dots) grouped genotypes with low‐medium hydroxycinnamic acid concentration, this group include commercial cultivars (ChA 0.77–3.14 g kg^−1^ DW). Cluster II (red dots) mainly grouped genotypes with medium‐high hydroxycinnamic concentration (ChA 0.88–4.96 g kg^−1^ DW). Cluster III (green dots) contains genotypes with high CaA (0.11–0.13 g kg^−1^ DW). Cluster IV (blue dots) mainly consisted of genotypes with the highest ChA and isomers concentrations (ChA 3.61–7.98 g kg^−1^ DW) Nariño landraces. Figure on the top right shows the direction vectors for the four hydroxycinnamic acids. ChA, chlorogenic acid; CaA, caffeic acid.

According to the results of the PCA, we chose the two principal components (PC) that explain 83.90% of the total variance, the PC1 (59.12%) and PC2 (24.78%) (Fig. 3). Within the factorial axes, the dominant traits in the first factorial axis are ChA and its isomers *neo*‐ChA and *crypto*‐ChA. These compounds showed similar vector directions indicating a strong relationship between these molecules, their loadings have a similar weight representing the similar contribution of these compounds in the total variation (PC1 eigenvectors: *crypto*‐ChA 0.59, ChA 0.56, and *neo*‐ChA 0.50). The relation of CaA with ChA and their isomers is observed in the second axis, the main contribution in the second factorial axis is given by CaA (PC2 eigenvector: CaA 0.87).

With a strong correlation between ChA, *crypto*‐ChA, and *neo*‐ChA compounds, which have similar vector directions, in contrast, CaA is negatively correlated with ChA and the isomers *crypto*‐ChA and *neo*‐ChA (Fig. 3). Our results are different to those reported by Andre et al. (2007) who analyzed these compounds in 23 native Andean cultivars. These authors found that ChA, *crypto*‐ChA, *neo*‐ChA, and CaA were located in the same PCA vector, which suggest a relationship between all of them. Localization in a factorial plane is associated mostly with average genotype concentration, their grouping is based on the main attributes, characterized by ChA, *crypto*‐ChA, and *neo*‐ChA variables.

Hierarchical clustering was constructed on two principal components in PCA, allowing the discrimination of different clusters using Euclidean distance as metric, Ward′s method as the agglomeration rule, and k‐means as algorithm for determining groups number (Fig. 3). Phureja genotypes were divided into four clusters using the main hydroxycinnamic compounds profiles (Table 1): Cluster I**,** composed of 48 genotypes with low to medium hydroxycinnamic acids concentrations; Cluster II**,** integrated by 51 genotypes with medium to high hydroxycinnamic acids concentrations; Cluster III, was the smallest group (three genotypes) and was characterized by genotypes with the highest CaA levels; Cluster IV, consisted of 11 genotypes with the highest concentrations of ChA, *neo*‐ChA, and *crypto*‐ChA.

ChA and its isomers are dietary desirable for their important effects in human health (Cho et al. 2010; Bassoli et al. 2008). The cluster analysis identified a group with high ChA and its isomers contents. Interestingly, six landraces from Nariño province were located in Cluster IV. The average ChA concentration (4.86 g kg^−1^ DW) found in our research is much more higher as compared to the contents on cultivars from the group Tuberosum, such as Russet cultivar (0.26 g kg^−1^ DW) or Victoria cultivar (0.25 g kg^−1^ DW) (Narváez‐Cuenca et al. 2012; Im et al. 2008).

In contrast, the principal Phureja commercial cultivars were grouped in the Cluster I, with the lowest hydroxycinnamic compounds concentration, including ChA. In our study we did not find specific groups according to flesh color as was found by other authors (Andre et al. 2007; André et al. 2009b). In our case red and purple genotypes were distributed in Clusters I and II, including the genotypes with low to medium‐high concentrations of hydroxycinnamic acids, therefore color cannot be related to a specific level hydroxycinnamic acids concentration (Table 1).

### Comparison between two agro‐climatic conditions

The hydroxycinnamic acids contents of each of the four compounds of eight genotypes were compared between two localities, Facatativá and Usme (2650 vs. 3400 masl, respectively) (Table 2) using the Wilcoxon test. Significant differences were found for *neo*‐ChA comparison (*P* = 0.002037) and for ChA comparison (*P* = 0.02867). The mean values for ChA of Usme increased twofold as compared to Facatativá (3.61 and 1.86 g kg^−1^ DW, respectively) and the mean values for *neo*‐ChA of Usme decreased twofold as compared to Facatativá (0.05 and 0.12, g kg^−1^ DW, respectively). Neither CaA nor *crypto*‐ChA contents were statistically affected by location. Although the experimental sample is small, it is possible to observe that the lower content of ChA in Facatativá locality is partly offset with a higher concentration of *neo*‐ChA. This result agrees with Payyavula et al. (2012), who reported the same effect using a commercial cultivar in extreme latitudes (67.2°, Alaska; 33.3°, Texas).

**Table 2:** Hydroxycinnamic compounds contents in cooked potato tubers (*Solanum tuberosum* group Phureja) cultivated in two localities with different altitudes

Localities								
Genotype	Usme (3400 masl)				Facatativá (2650 masl)			
	Chlorogenic acid (ChA)	*neo*‐ChA	*crypto*‐ChA	Caffeic acid	Chlorogenic acid (ChA)	*neo*‐ChA	*crypto*‐ChA	Caffeic acid
	g kg^−1^ DW				g kg^−1^ DW			
CCC037	5.46 ± 0.87	0.03 ± 0.01	0.58 ± 0.10	0.03 ± 0.01	2.42 ± 0.37	0.23 ± 0.06	0.75 ± 0.16	0.06 ± 0.03
CCC067	1.78 ± 0.41	0.02 ± 0.01	0.27 ± 0.06	0.04 ± 0.01	1.61 ± 0.18	0.08 ± 0.05	0.40 ± 0.06	0.03 ± 0.02
CCC101	1.29 ± 0.03	0.04 ± 0.00	0.15 ± 0.00	0.04 ± 0.00	1.11 ± 0.45	0.04 ± 0.03	0.17 ± 0.04	0.02 ± 0.00
CCC102	6.07 ± 0.81	0.16 ± 0.01	1.11 ± 0.15	0.06 ± 0.01	2.20 ± 0.33	0.26 ± 0.16	0.99 ± 0.26	0.05 ± 0.02
CCC110	8.40 ± 1.98	0.05 ± 0.01	0.80 ± 0.18	0.07 ± 0.01	2.68 ± 1.66	0.12 ± 0.11	0.44 ± 0.16	0.08 ± 0.10
CCC133	2.44 ± 0.17	0.05 ± 0.00	0.03 ± 0.02	0.03 ± 0.00	1.44 ± 0.73	0.05 ± 0.05	0.34 ± 0.30	0.03 ± 0.03
CCC136	2.34 ± 0.20	0.05 ± 0.00	0.34 ± 0.00	0.10 ± 0.01	1.98 ± 0.59	0.10 ± 0.04	0.40 ± 0.17	0.05 ± 0.01
CCC144	1.13 ± 0.07	0.03 ± 0.00	0.18 ± 0.01	0.01 ± 0.00	1.44 ± 1.05	0.06 ± 0.04	0.22 ± 0.14	0.03 ± 0.02
Mean	3.61	0.05**	0.4	0.05	1.86**	0.12**	0.46	0.04

Data are presented as mean (*n* = 3) ± SD. ChA, chlorogenic acid; CaA, caffeic acid. Asterisks indicate statistic significance.

*P <* *0.05.

**P < 0.01.

In conclusion, potato tubers group Phureja presented a wide variation in hydroxycinnamic acids concentrations, reflecting high genetic diversity among potato genotypes. These genetic materials can be exploited in breeding to improve hydroxycinnamic acid contents, especially Nariño landraces with high ChA concentration (up to 7.98 g kg^−1^ DW), thus contributing to human health protection.

## Conflict of Interest

The authors of this publication declare that there is no conflict of interest for any aspect.
